# COVID-19 Knowledge, Anxiety, and Access to Voluntary Counseling and Testing Among People With HIV During the COVID-19 Pandemic: Cross-Sectional Study

**DOI:** 10.2196/75227

**Published:** 2026-06-04

**Authors:** Sitti Shoimatul Azizah, Sri Yona, Risyda Zakiyah Hanim, Rahmat Kurniawan, Anggri Noorana Zahra

**Affiliations:** 1 Department of Nursing, Nursing Ward, Anggrek, RSUD Abdoel Wahab Sjahranie Samarinda, Samarinda, Indonesia; 2 Faculty of Nursing, University of Indonesia, Jl. Prof. Dr. Bahder Djohan, Kampus UI Depok, Depok, West Java, 16424, Indonesia, 62 81293912127

**Keywords:** COVID-19 knowledge, voluntary counseling testing, anxiety, health service, people living with HIV, antiretroviral therapy, ART

## Abstract

**Background:**

The COVID-19 pandemic caused significant disruption in health care services. Many essential services were delayed by health care facilities, including voluntary counseling and testing (VCT) services for people living with HIV. There were many reports of interruption in HIV testing, antiretroviral therapy (ART) initiation, and also ART access for people living with HIV during the pandemic. Patients were unable to attend follow-ups and acute care visits due to fear and anxiety. This situation also caused stress for people living with HIV.

**Objective:**

This study aimed to determine the level of COVID-19 knowledge, anxiety, and access to VCT services for people living with HIV.

**Methods:**

This cross-sectional, correlational study was conducted with 200 participants at 1 public hospital in Samarinda (n=140, 70%) and 1 public hospital in South Jakarta (n=60, 30%), Indonesia, from August 2022 to April 2023. Sampling was done using convenience methods and predefined inclusion criteria. Data collection included a demographic information form, COVID-19 knowledge questionnaire, the Coronavirus Anxiety Scale, and a questionnaire assessing access to health services.

**Results:**

Both COVID-19 knowledge (odds ratio 11.246, 95% CI 11.246; *P*=.001) and anxiety (odds ratio 2.258, 95% CI 2.216; *P*=.03) had a positive and significant relationship with access to health services. A multivariate analysis showed that the most influential factor affecting access to VCT services was knowledge (*P*=.001; B=2.289).

**Conclusions:**

This study highlights the need for enhanced support and education for people living with HIV or AIDS regarding their knowledge of and anxiety related to COVID-19, particularly considering their vulnerabilities. To ensure compliance with health protocols in future pandemics, it is crucial to improve access to health care services. One key recommendation is to enhance the VCT service system, especially for people living with HIV or AIDS, during public health emergencies such as the COVID-19 pandemic. Additionally, services such as telemedicine and telehealth should be further developed to allow people living with HIV or AIDS to receive ART without the need for in-person hospital visits.

## Introduction

HIV is a significant global public health issue. People living with HIV require long-term treatment to achieve and maintain a good quality of life. The COVID-19 pandemic posed substantial global public health challenges and disrupted HIV testing and reporting worldwide [1]. In 2023, approximately 42.3 million people were living with HIV globally, of whom 28.9 million were receiving antiretroviral therapy (ART). These individuals require regular clinical monitoring and follow-up in HIV health care settings. However, during the pandemic, health care services were disrupted, including those serving people living with HIV. Access to HIV testing services was limited during the pandemic, and many people living with HIV were reluctant to visit health care facilities due to fear of COVID-19 infection [2]. People with chronic health conditions, such as HIV, may experience increased stress because of their heightened vulnerability to COVID-19 [3]. A study conducted in Hong Kong found that reduced contact with the LGBT (lesbian, gay, bisexual, and transgender) community during the COVID-19 pandemic was strongly associated with difficulties in accessing HIV services [4]. Several studies have highlighted the negative impact of the COVID-19 pandemic on HIV services for people living with HIV [4-6]. For example, a qualitative study in southwest Uganda [6] reported that COVID-19–related restrictions prevented patients from accessing HIV testing services. The pandemic also disrupted efforts to control the HIV epidemic by affecting the management of HIV care and continuity of access to ART. Concerns were raised regarding challenges in ART initiation and retention in care, fear of COVID-19 exposure, ART supply chain disruptions, and the potential increase in HIV incidence due to secondary transmission from individuals with detectable HIV loads [7]. Furthermore, the provision of HIV services to socially marginalized groups was particularly affected, exacerbating existing vulnerabilities.

During pandemics, access to health care services is often compromised. Even prior to the COVID-19 pandemic, factors such as stigma, acceptance of lifelong treatment, financial constraints related to distance and transportation, gender, and education level influenced access to voluntary counseling and testing (VCT) services [8]. Several studies have reported that COVID-19 disrupted HIV services, including HIV testing and access to ART medications. This disruption is particularly concerning because people living with HIV require continuous follow-up care and consistent ART to suppress viral replication and maintain viral load suppression. Therefore, ensuring uninterrupted access to ART remains essential [9-11]. The aim of this study was to examine the relationship between COVID-19 knowledge, anxiety, and access to VCT among people living with HIV during the COVID-19 pandemic.

## Methods

### Study Design, Population, and Sample

This study used a cross-sectional survey design and was conducted at 2 HIV outpatient hospitals: 1 in Jakarta and 1 in Samarinda, East Kalimantan, Indonesia. These public hospitals offer comprehensive HIV services, including HIV testing, ART, and VCT services for people living with HIV. Data collection was carried out from July 2022 to April 2023. The minimum required sample size was 140 (using the Slovin formula); however, 200 respondents were successfully recruited. The eligibility criteria are shown in Textbox 1.

Textbox 1. **Participant eligibility criteria.**

**Inclusion criteria**

- Confirmed diagnosis of HIV
- Aged ≥18 years
- Ability to provide written responses to the study questionnaire
- Adequate level of consciousness and responsiveness as assessed by the Glasgow Coma Scale (GCS)

**Exclusion criteria**

- Individuals without an HIV diagnosis
- Aged <18 years
- Individuals unable to provide written responses
- Those who were not adequately responsive based on the GCS assessment

### Data Collection Tools

The instruments used in this study included a COVID-19 knowledge questionnaire [12], the Coronavirus Anxiety Scale [13], and a questionnaire assessing access to VCT services [14]. In addition, a demographic questionnaire was used to collect data on participants’ characteristics, including age, gender, marital status, education level, place of residence, and COVID-19 infection status.

The COVID-19 knowledge questionnaire assessed participants’ knowledge and attitudes toward COVID-19 using a 5-point Likert scale. For each of the 6 statements, respondents indicated their level of agreement as “strongly disagree,” “disagree,” “undecided,” “agree,” or “strongly agree.” The final section of the questionnaire assessed preventive practices through 5 items related to behaviors such as (1) attending large social gatherings; (2) visiting crowded places; (3) avoiding cultural practices such as handshaking; (4) practicing social distancing; and (5) washing hands after sneezing, coughing, blowing the nose, or being in public places.

The Coronavirus Anxiety Scale consists of 5 items measuring anxiety symptoms related to COVID-19. Respondents rated how frequently they experienced each symptom over the past 2 weeks using a scale ranging from “not at all” to “almost every day.” The items assessed symptoms such as (1) feeling dizzy, lightheaded, or faint when exposed to news about COVID-19; (2) difficulty falling or staying asleep due to thoughts about COVID-19; (3) feeling paralyzed or frozen when exposed to information about COVID-19; (4) loss of appetite when thinking about COVID-19; and (5) nausea or stomach discomfort triggered by thoughts about COVID-19.

The access to VCT services questionnaire was modified to include 7 items measured using a Guttman scale with dichotomous (yes or no) response options. The items assessed access-related experiences during the pandemic, including routine VCT service visits, difficulty finding time to collect medication, distance barriers, the ability to obtain medication through alternative means (eg, online or delivery services), financial constraints, increased treatment costs, and fear of stigma when visiting VCT services.

### Statistical Analysis

All data were edited, coded, and entered into SPSS (version 25; IBM Corp) for analysis. Descriptive statistics were used to summarize respondents’ demographic characteristics. Univariate analysis was conducted for all variables to examine frequency distributions and percentages. Before carrying out bivariate analysis, a normality test was performed on the independent and confounding variables. The Kolmogorov-Smirnov test was applied because the sample size exceeded 50 (N=200). The results indicated that the data were normally distributed (*P*>.05). Bivariate analysis was performed using the chi-square test to examine the relationships between the study variables. Subsequently, multivariate analysis was conducted using logistic regression to identify the variables that exerted the most significant influence on the dependent variable. The level of statistical significance was set at 95% CI (α=.05). A *P* value of <.05 was considered statistically significant, leading to rejection of the null hypothesis (H_0_), whereas a *P* value >.05 indicated failure to reject the null hypothesis.

### Ethical Considerations

Ethics approval for this study was obtained from the Ethics Committee of the Faculty of Nursing, University of Indonesia (KET-146/UN2.F12.2.1/PPM.00.02/2023) and from the Ethics Committee of the Hospital in Samarinda (362/KEPK-AWS/X/2021). Participation was voluntary. Prior to participation, all respondents provided written informed consent. Participants were provided with a written information sheet explaining the study procedures, objectives, potential benefits, possible risks, and their rights and obligations as research participants.

## Results

### Characteristics of Respondents

Table 1 shows the sociodemographic characteristics of the respondents and their access to voluntary counseling and HIV testing. A majority of the respondents were men (n=122, 61%) and were aged <40 years (146/200, 73%). Regarding educational attainment, 103 (51.5%) respondents had completed high school. Additionally, 54% (n=108) of the respondents were married and resided in Samarinda. More than 80% (n=160) of participants had never been diagnosed with COVID-19. No significant associations were found between respondents’ demographic characteristics and access to VCT services.

**Table 1.** Correlation between respondent characteristics based on age, gender, education, marital status, domicile, COVID-19 infection, and access to voluntary counseling and testing (VCT; N=200).

Characteristics	Access to VCT		
	Respondents, n (%)	Odds ratio (95% CI)	*P* value
Age (years)		0.572 (0.302‐1.086)	.11
<40	146 (73)		
>40	54 (27)		
Gender		0.869 (0.491‐1.537)	.73
Man	122 (61)		
Woman	78 (39)		
Education		0.757 (0.308‐1.862)	.70
High (senior high school-university)	22 (11)		
Low (elementary-high school)	178 (89)		
Marital status		1047 (0.592‐1.851)	.99
Single	77 (38.5)		
Married	108 (54)		
Divorced	15 (7.5)		
Domicile		0.18 (0.004‐0.077)	.001^a^
Samarinda	140 (70)		
Jakarta	60 (30)		
COVID-19 infection		0.47 (0.226‐0.978)	.06
Yes	37 (18.5)		
No or never	163 (81.5)		

a *P*<.05.

### Level of Knowledge and Attitudes

Table 2 reports COVID-19 knowledge and anxiety in the respondents. More than half of the respondents demonstrated a high level of COVID-19 knowledge (112/200, 56%). The majority of respondents reported low levels of anxiety related to COVID-19 (122/200, 61%). A slight majority (106/200, 53%) of respondents indicated that their access to VCT services was good.

**Table 2.** Frequency distribution based on COVID-19 knowledge and anxiety in people living with HIV.

Variables	Access to VCT^a^ services		*P* value
	Respondents, n (%)	Odds ratio (95% CI)	
COVID-19 knowledge		11.246 (5789-21.848)	.001^b^
High	112 (56)		
Low	88 (44)		
Anxiety		2.216 (1.242‐3.955)	.01^b^
No	122 (61)		
Yes	78 (39)		

a VCT: voluntary counseling and testing.

b *P*<.05.

### Findings of the Logistic Regression Analysis

Table 3 shows that among the variables examined, only COVID-19 knowledge and COVID-19 anxiety were significantly associated with access to VCT services. COVID-19 knowledge was identified as the most influential factor affecting access to VCT services.

**Table 3.** The relationship among variables with access to voluntary counseling and testing services.

Research variables	B	*P* value	Odds ratio (95% CI)
Age (years)	−0.245	.56	0.783 (0.341‐1.798)
Gender	0.156	.70	1169 (0.522‐2.617)
Education	−0.266	.64	0.767 (0.252‐2.335)
COVID-19 status	−0.514	.25	0.598 (0.247‐1.446)
Marital status	−0.421	.25	0.656 (0.317‐1.358)
COVID-19 knowledge	2289	.001^a^	9.861 (4.916‐19.778)
COVID-19 anxiety	0.815	.02	2258 (1.106‐4.612)

a *P*<.05.

## Discussion

### Principal Findings

The results of this study demonstrated a significant association between COVID-19 knowledge and access to VCT services (*P*=.001). This finding indicates that higher levels of COVID-19 knowledge are associated with improved access to comprehensive health care services, including VCT services. Although limited studies have specifically examined the relationship between COVID-19 knowledge and access to VCT services, previous research has identified several contributing factors to the use of VCT services, including adequate knowledge of HIV, strong motivation to maintain health, and psychological responses such as stress and anxiety related to COVID-19. In addition, increased levels of depression and anxiety and disruptions in health care access were reported during the pandemic, particularly among women with chronic illnesses and ethnic minority groups who experienced significantly more appointment cancellations during lockdown periods [15]. Similarly, a study by Sitopu and Ndrudu [16] in Medan reported that only 50.6% of patients with good knowledge accessed VCT services.

Knowledge is a critical determinant of health-related behavior, including the use of VCT services [17]. People with adequate knowledge of COVID-19 may be more proactive in seeking HIV-related services to maintain their health. In this study, respondents with lower levels of anxiety were more likely to access VCT services. This may be associated with adequate COVID-19 knowledge, which could reduce anxiety levels. More than half of the respondents demonstrated adequate knowledge regarding preventive measures against COVID-19, such as wearing masks, frequent handwashing, and practicing social distancing. Additionally, information regarding COVID-19 was widely disseminated via social media platforms, such as WhatsApp and Instagram, increasing public access to health information.

During the COVID-19 pandemic, health care systems were significantly disrupted. The crisis exacerbated existing social inequalities and shifted health care priorities toward the treatment of patients with COVID-19. Consequently, people living with HIV faced barriers to accessing essential services, including VCT. These findings highlight the need for greater attention to the use of mass media, both print and electronic, during the COVID-19 pandemic. It is essential to disseminate information about HIV or AIDS services and VCT service access, as well as to organize seminars and workshops on these topics. These initiatives can help increase knowledge related to HIV or AIDS. Furthermore, additional studies should analyze other potential factors influencing individuals’ willingness to participate in VCT. Identifying stronger predictors of knowledge, such as stigma toward HIV/AIDS, can provide valuable insights and create greater opportunities for intervention. By addressing these factors, we can develop targeted strategies to overcome the challenges faced in accessing HIV services [18].

In a survey conducted among residents of Wuhan, China, during the early COVID-19 outbreak (January 31-February 2, 2020), 17% of respondents reported experiencing moderate to severe depression, while 29% reported moderate to severe anxiety [19]. People living with HIV expressed anxiety during the pandemic regarding their treatment processes, particularly concerning the availability of ART and VCT services. This concern is significant because the immune systems of people living with HIV, which may not have improved, could have increased their risk of illness. However, it is important to note that there is no evidence indicating an increased risk of COVID-19 in people living with HIV [20].

Importantly, in this study, most of the participants reported low levels of anxiety regarding COVID-19. This suggests that adequate knowledge about COVID-19 may lead to reduced symptoms of anxiety. Consequently, this can increase access to VCT services during the pandemic. A study by Jayani and Eureka [21] on 34 people living with HIV showed a significant relationship between the level of knowledge and the level of stress experienced during the COVID-19 pandemic. Strengthening knowledge by providing education to people living with HIV throughout the pandemic was essential for this at-risk group, not only in relation to adherence to ART but also for complying with health protocols during the pandemic [21].

The anxiety experienced by people living with HIV during the COVID-19 pandemic is consistent with previous research [3], which found that people living with HIV experience anxiety and depression due to their illness, as they are at risk of death because of low immunity. This condition is further exacerbated by the COVID-19 pandemic, during which people living with HIV are at a higher risk of contracting the virus. Research findings also indicate that government-imposed restrictions disrupted treatment services. Although health care providers offered remote services in the form of telehealth, many people living with HIV were constrained by limited resources.

Psychological pressure during the COVID-19 pandemic was also experienced by people living with HIV. In addition to facing social stigma, they struggled to live in uncertain circumstances caused by the pandemic [19]. One of the most effective interventions to reduce anxiety is coping through social support, such as support from a partner or family members [18]. This finding is consistent with the results of this study, in which the majority of respondents received support from their family. Spouses also played an important role in assisting with ART collection at health facilities, reducing the need for people living with HIV to leave their homes during the COVID-19 pandemic [22]. Married people living with HIV tend to receive positive social support from their partners, which helps them cope with the challenges of a pandemic. The anxiety experienced by people living with HIV regarding their health was intensified by the COVID-19 pandemic, during which they were considered a high-risk group due to compromised immune systems. It can therefore be concluded that anxiety related to COVID-19 can influence people living with HIV in accessing VCT services because of their perceived vulnerability to illness. It is recommended that VCT service providers enhance and expand access to services for people living with HIV and address resource-related barriers to ensure continuity of treatment and maintain their quality of life [23].

### Implications and Limitations

Adequate knowledge about COVID-19 and reduced symptoms of anxiety during the pandemic were significantly associated with access to VCT services. It is essential to integrate COVID-19 health education with social media platforms such as WhatsApp and Instagram to increase awareness. This approach may enable people living with HIV to access health care services with less concern about contracting COVID-19. Furthermore, similar strategies can be applied to various health topics to enhance the quality of life of people living with HIV. Our sample was relatively younger and more likely to be married. Although this may limit the generalizability of the findings to older people living with HIV in Indonesia, the results suggest that individuals with higher education, younger age, and greater exposure to information through social media tend to experience lower levels of anxiety and demonstrate better health outcomes.

### Conclusions

This study highlights the need for enhanced support and education for people living with HIV or AIDS regarding their knowledge of and anxiety related to COVID-19, particularly considering their vulnerabilities. To ensure compliance with health protocols in future pandemics, it is crucial to improve access to health care services. One key recommendation is to enhance the VCT service system, especially for people living with HIV or AIDS, during public health emergencies such as the COVID-19 pandemic. Additionally, services such as telemedicine and telehealth should be further developed to allow people living with HIV or AIDS to receive ART without the need for in-person hospital visits.

Furthermore, VCT service providers should offer targeted education and support to individuals who experience difficulties in accessing health care services. Addressing the challenges faced by people living with HIV or AIDS, particularly those arising from anxiety related to the COVID-19 pandemic, is crucial to ensuring continuity of care and improving overall well-being.

## Abbreviations

ART: antiretroviral therapy
LGBT: lesbian, gay, bisexual, and transgender
VCT: voluntary counseling and testing
